# Investigation of a Photoelectrochemical Passivated ZnO-Based Glucose Biosensor

**DOI:** 10.3390/s110504648

**Published:** 2011-04-28

**Authors:** Ching-Ting Lee, Ying-Shuo Chiu, Shu-Ching Ho, Yao-Jung Lee

**Affiliations:** 1 Institute of Microelectronics, Department of Electrical Engineering, National Cheng Kung University, 701, Tainan, Taiwan; E-Mail: q18991120@mail.ncku.edu.tw (Y.-S.C.); 2 Microsystems Technology Center, Industrial Technology Research Institute, Tainan, Taiwan; E-Mails: itri990067@itri.org.tw (S.-C.H.); yjlee@itri.org.tw (Y.-J.L.)

**Keywords:** extended-gate field-effect-transistors, photoelectrochemical method, vapor cooling condensation technique, ZnO-based glucose biosensors, ZnO nanorods

## Abstract

A vapor cooling condensation system was used to deposit high quality intrinsic ZnO thin films and intrinsic ZnO nanorods as the sensing membrane of extended-gate field-effect-transistor (EGFET) glucose biosensors. The sensing sensitivity of the resulting glucose biosensors operated in the linear range was 13.4 μA mM^−1^ cm^−2^. To improve the sensing sensitivity of the ZnO-based glucose biosensors, the photoelectrochemical method was utilized to passivate the sidewall surfaces of the ZnO nanorods. The sensing sensitivity of the ZnO-based glucose biosensors with passivated ZnO nanorods was significantly improved to 20.33 μA mM^−1^ cm^−2^ under the same measurement conditions. The experimental results verified that the sensing sensitivity improvement was the result of the mitigation of the Fermi level pinning effect caused by the dangling bonds and the surface states induced on the sidewall surface of the ZnO nanorods.

## 1. Introduction

Recently, there has been great concern about health issues due to the irregular living and diet habits of humans. Diabetes mellitus is one of the main causes of death and disability. It can cause heart disease, kidney nephropathy, and blindness. Glucose biosensors, which possess high sensitivity, low-cost, reliable characteristics for biomedical measurement, have become key instruments in blood glucose monitors, biological analyses, chemical analyses, and clinical detection. Field-effect-transistor (FET) -based devices have been widely used in biosensors [1,2]. Among the FET-based biosensors, the extended-gate field-effect-transistor (EGFET) is a promising device thanks to its easier fabrication process [3]. The extended-gate field-effect-transistors consist of two parts, including the sensing membrane structure and the metal-oxide-semiconductor field-effect-transistor (MOSFET) structure. With its isolated electrical and sensing parts, the MOSFET does not need to be in contact with the solutions during the biosensor measurement process. Moreover, the extended-gate sensing electrode is less influenced by the optical illumination and the operation temperature. Besides, it can be disposable. Therefore, biosensors of EGFET structure can be applied in fast, convenient and low cost medical tests.

In view of its high stability and selectivity to glucose, glucose oxidase (GOD) has been widely utilized in glucose biosensors, especially the amperometric glucose biosensors [4]. To establish a friendly environment for immobilizing the enzyme, the sensing membrane of glucose sensors has to be selected appropriately. ZnO is a promising membrane material due to its wide bandgap (3.3 eV), stability, non toxicity, and ideal biocompatible properties. Furthermore, because the isoelectric point (IEP) of ZnO is about 9.5, the ZnO is suitable for adsorption of low IEP proteins or enzyme [5]. In view of these advantages of high specific surface area, good biological compatibility and stability, ZnO nanorods have been attracted intense attention and have been used in various biosensors [3,5–7]. Although several methods have been developed to deposit ZnO-based materials onto various substrates [8–11], the high quality and high resistivity intrinsic ZnO film and intrinsic ZnO nanorods required in glucose biosensors are difficult to obtain, because the ZnO usually exhibits n-type conductivity behavior owing to the compensation effect induced by the oxygen vacancies and the zinc interstitials [12,13]. Recently, a novel vapor cooling condensation system was built to deposit high quality ZnO films and ZnO nanorods [14,15]. In this work, in order to fabricate the sensing membrane part of the EGFETs, ZnO films and nanorods were deposited using this vapor cooling condensation system. However, a Fermi level pinning effect is induced by the existence of a lot of dangling bonds and surface states located on the sidewall surface of the ZnO nanorods. Consequently, the band alignment of the electrolyte/semiconductor junction cannot be effectively changed with the various pH values of the measuring solution. Therefore, the sensing performance of the glucose biosensors is seriously degraded. In this work, to circumvent this drawback, the sidewall surface of the ZnO nonorods was passivated using the photoelectrochemical (PEC) method. Consequently, the influence of the Fermi level pinning effect can be mitigated owing to the reduction of the dangling bonds and the surface states.

## 2. Experimental Process

**Figure 1.**
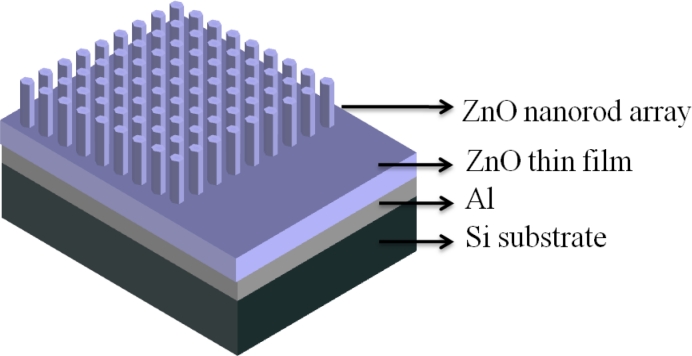
The schematic configuration of ZnO nanorod glucose biosensors.

To fabricate the glucose biosensor EGFETs, a 100-nm-thick Al layer was first deposited on a silicon (Si) substrate as the conducing layer. A 200-nm-thick intrinsic ZnO film and an 80-nm-long intrinsic ZnO nanorod array were then deposited on the Al conducting layer using the vapor cooling condensation system. A schematic configuration of the ZnO glucose biosensors is shown in Figure 1.

The electron concentration and electron mobility of the deposited intrinsic ZnO films were 2.3 × 10^15^ cm^−3^ and 3.1 cm/V-sec, respectively. For depositing the nanorod array, an anodic alumina membrane (AAM) template was intimately covered with the deposited intrinsic ZnO film. The pore diameter and pore density of the AAM template were 100 nm and 5 × 10^9^ cm^−2^, respectively. During the deposition of the intrinsic ZnO film and nanorod array, the Si substrate was attached on a liquid nitrogen-cooled stainless steel plate. The sublimated ZnO vapor materials originated from the heated ZnO powder (purity = 99.99%) loaded in a tungsten boat were then cooled and condensed on the Si substrate. To passivate the dangling bonds and the surface states induced on the sidewall surface of the intrinsic ZnO nanorods using the photoelectrochemical method, a thin Zn(OH)_2_ layer was directly grown in an ammonia (NH_3_) chemical solution (pH value = 8) under the illumination of a He-Cd laser (power density = 10.0 mW/cm^2^ and wavelength = 325 nm). This photoelectrochemical system was reported previously [16,17]. The reactions taking place in the photoelectrochemical process can be described as follows:

(1) $${\text{NH}}_{3}\;+\;{\text{H}}_{2}\text{O}\;\to \;{\text{NH}}_{4}^{+}\;+\;{\text{OH}}^{-}$$

(2) $$\text{Zn}\;+\;{2\text{h}}^{+}\;+\;2{\text{OH}}^{-}\;\to \;{\text{Zn(OH)}}_{2}$$

where h^+^ represents the holes optically generated by the illumination with the He-Cd laser. A thin Zn(OH)_2_ layer was formed on the sidewall surface of the intrinsic ZnO nanorods and passivated the dangling bonds and the surface states. To fabricate the glucose biosensors, the sensing membrane of the EGFETs was encapsulated with an epoxy resin and only the exposed active sensing region was left. Furthermore, for fabricating the enzyme immobilization layer, a 3-glycidyloxypropyltrimethoxysilane (GPTS) and toluene mixture was spun on the sensing membrane. After baking at 120 °C for two hours, the biosensor was immersed in a phosphate buffer solution (PBS) (5 mM, pH = 7) to wash away the unbounded GPTS. After the mixture solution of GOD (100 U/mg) and 5 mM phosphate buffer solution (pH value of 7.4) was dropped onto the enzyme immobilization layer, the biosensor was shielded from light and kept at 4 °C for 24 h.

## 3. Experimental Results and Discussion

### 3.1. Measurement System

**Figure 2.**
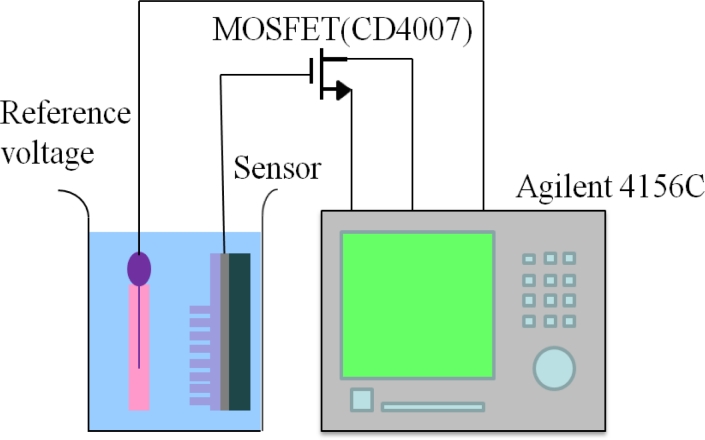
The measurement system of ZnO nanorod glucose biosensors.

To measure the pH sensing characteristics of the glucose biosensors, the biosensor and a Ag/AgCl reference electrode were dipped into the buffer solution (the pH value varied from 4 to 12) and the Al conducting layer was connected to the gate of the commercial MOSFET device (CD4007). An Agilent 4156C Semiconductor Parameter Analyzer was used to measure the drain-source current of the MOSFET device. The measuring system is shown in Figure 2.

### 3.2. pH Sensing Characteristic

**Figure 3.**
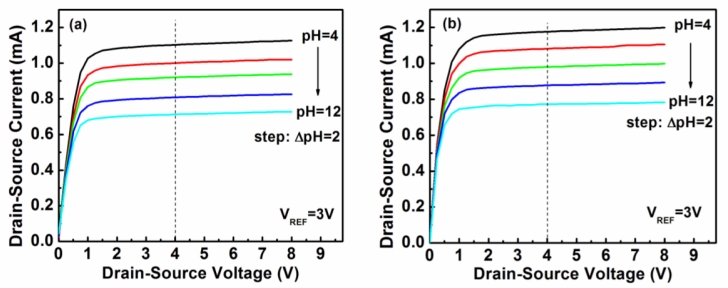
Drain-source current—drain-source voltage characteristics of the glucose biosensors with **(a)** unpassivated ZnO nanorod, and **(b)** passivated ZnO nanorod.

The dependence of the drain-source current (I_DS_) on the drain-source voltage (V_DS_) of the glucose biosensors with unpassivated ZnO nanorods and with passivated ZnO nanorods operated at a reference electrode voltage (V_REF_) of 3 V is shown in Figure 3(a,b), respectively.

**Figure 4.**
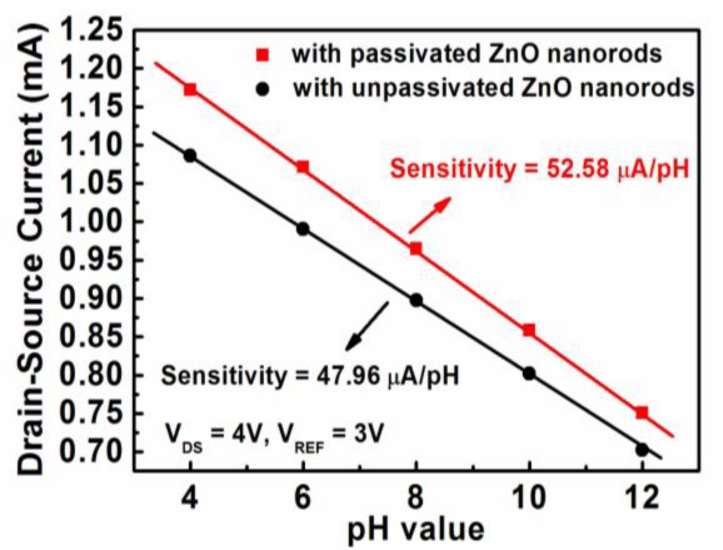
Drain-source current as a function of pH value.

Based on the experimental results shown in Figure 3(a,b), the measured drain-source current as a function of the pH value of the two kinds of biosensors is shown in Figure 4, where the drain-source voltage (V_DS_) of the MOSFET operated at 4 V and the reference electrode voltage (V_REF_) was 3 V. The pH sensitivity shown in Figure 4 was calculated from the linear relation between the drain-source current and the pH value of the two kinds of biosensors. The sensing sensitivity of the glucose biosensors with unpassivated and passivated ZnO nanorods was 47.96 μA/pH and 52.58 μA/pH, respectively. It could thus be seen that the passivation of the nanorods improved the sensing sensitivity of the glucose biosensors. This experimental result verified that the glucose biosensors with PEC passivation treatment have better sensing performances due to the reduction of Fermi level pinning effect caused by the dangling bonds and the surface states.

### 3.3. The Sensing Characteristics of Glucose Biosensors

The mechanism of electrochemical glucose biosensors is based on an enzymatic reaction catalyzed by GOD according to the following reaction [18]:

(3) $$\beta \text{-D-glucose}\;+\;{\text{O}}_{2}\;\overset{\text{GOD}}{\to }\;\text{D-glucono-δ-lactone}\;+\;{\text{H}}_{2}{\text{O}}_{2}$$

(4) $$2{\text{H}}_{2}{\text{O}}_{2}\;\to \;2{\text{H}}_{2}\text{O}\;+\;{\text{O}}_{2}$$

(5) $$\text{D-glucono-δ-lactone}\;+\;{\text{H}}_{2}\text{O}\;\to \;\text{D-gluconate}\;+\;{\text{H}}^{+}$$

**Figure 5.**
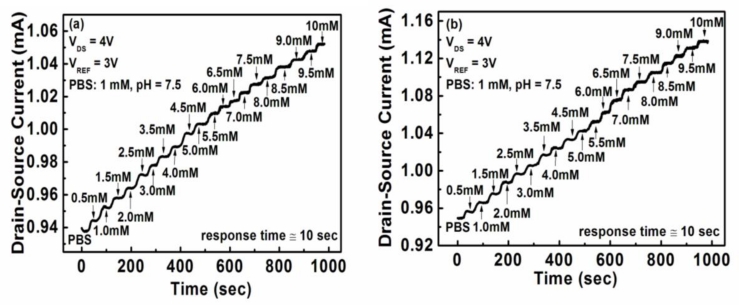
Drain-source current response of **(a)** the unpassivated ZnO nanorod glucose biosensors and **(b)** the passivated ZnO nanorod glucose biosensors.

In the reaction of Equation (3), the β-d-glucose was catalyzed by the glucose oxidase to produce hydrogen peroxide and d-glucono-δ-lactone. The hydrogen peroxide was then spontaneously converted to H_2_O and oxygen as indicated in Equation (4). The oxygen was further reused in the reaction of glucose catalyzing. Finally, the d-glucono-δ-lactone was hydrolyzed to gluconic acid and produced hydrogen ions (H^+^) as shown in the reaction of Equation (5). The production of hydrogen ions reduced the pH value of the medium. Consequently, the glucose concentration could be determined by the change of pH value. To measure the sensing sensitivity and the response speed, the glucose biosensor was immersed in 1 mM phosphate buffer solution (PBS) (pH = 7.5) and the glucose concentration gradually increased in 0.5 mM steps. Figure 5(a,b) show the drain-source current response of the unpassivated and the passivated ZnO nanorod glucose biosensors operated at a drain-source voltage (V_DS_) of 4 V and a reference electrode voltage (V_REF_) of 3 V, respectively.

**Figure 6.**
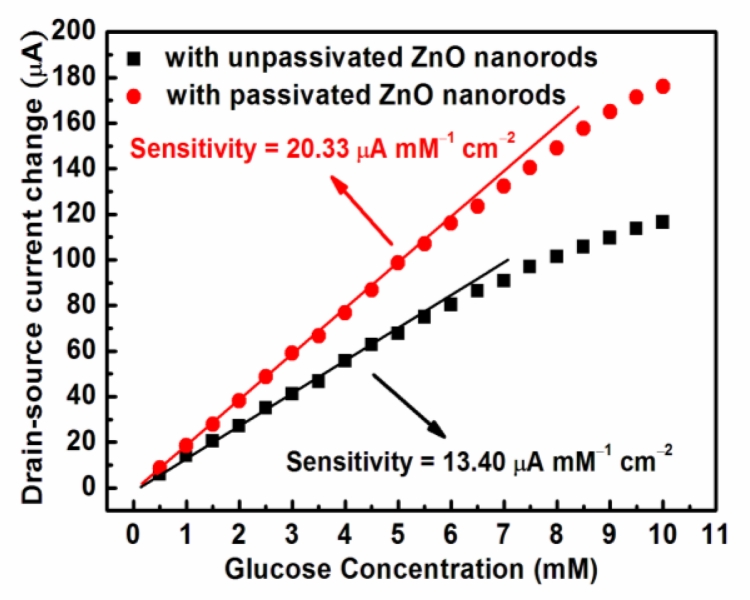
Drain-source current change as a function of glucose concentration of the unpassivated and the passivated glucose biosensors.

Based on the experimental results shown in Figure 5(a,b), the drain-source current change as a function of the glucose concentration is shown in Figure 6. The drain-source current change was referred the associated current measured in PBS solution. Both the unpassivated and the passivated ZnO nanorod glucose biosensors exhibited linear sensing sensitivity at a lower glucose concentration and then were prone to gradual saturation at a higher glucose concentration. The response time was about 10 s. The linear sensing sensitivity of the unpassivated and the passivated ZnO nanorod glucose biosensors was 13.4 μA mM^−1^ cm^−2^ and 20.33 μA mM^−1^ cm^−2^, respectively.

## 4. Conclusions

In this work, a vapor cooling condensation system was successfully utilized to deposit the high quality intrinsic ZnO film and nanorods required for the fabrication of EGFETs in glucose biosensors. A sensing sensitivity of 13.4 μA mM^−1^ cm^−2^ was obtained with the resulting glucose biosensors. To improve the sensing performance, a photoelectrochemical method was used to passivate the dangling bonds and the surface states located at the sidewall surface of the ZnO nanorods The Fermi level pinning effect was thus diminished by the passivation and the sensitivity of the passivated ZnO nanorod glucose biosensors was improved to 20.33 μA mM^−1^ cm^−2^. These promising ZnO-based biosensors can be expected to be applied in biomedical instruments by using the developed vapor cooling condensation system and the photoelectrochemical method.
